# The Heparin-Binding Hemagglutinin of *Nocardia cyriacigeorgica* GUH-2 Stimulates Inflammatory Cytokine Secretion Through Activation of Nuclear Factor κB and Mitogen-Activated Protein Kinase Pathways via TLR4

**DOI:** 10.3389/fcimb.2020.00003

**Published:** 2020-02-13

**Authors:** Xingzhao Ji, Xiujuan Zhang, Lina Sun, Xuexin Hou, Han Song, Lichao Han, Shuai Xu, Heqiao Li, Xiaotong Qiu, Minghui Li, Xuebing Wang, Ningwei Zheng, Zhenjun Li

**Affiliations:** ^1^State Key Laboratory for Infectious Disease Prevention and Control, National Institute for Communicable Disease Control and Prevention, Chinese Center for Disease Control and Prevention, Beijing, China; ^2^Department of Endocrinology, Beijing Chaoyang Hospital, Capital Medical University, Beijing, China; ^3^School of Laboratory Medicine and Life Sciences, Wenzhou Medical University, Wenzhou, China; ^4^Department of Medicine, Tibet University, Lhasa, China; ^5^CAS Key Laboratory of Pathogenic Microbiology and Immunology, Institute of Microbiology, Chinese Academy of Sciences, Beijing, China

**Keywords:** *Nocardia cyriacigeorgica*, HBHA, adhesion, cytokines, MAPK, NF-κB

## Abstract

Heparin-binding hemagglutinin (HBHA) from mycobacteria is involved in the dissemination of infection and the activation of the host immune response. However, the interaction of *Nocardia cyriacigeorgica* HBHA with the host cells remains unknown. In the present study, we describe *N. cyriacigeorgica* HBHA interactions with epithelial cells and organ colonization. We then investigate the mechanisms by which HBHA induces the production of inflammatory cytokines in macrophages. Immunofluorescent microscopy showed that HBHA adhered to A549 cells and HeLa cells and that the C-terminal fragment, which contains a Pro-Ala-Lys–rich domain, was responsible for adhesion. The deletion of the *hbha* gene in *N. cyriacigeorgica* mutant strains impaired adhesion to A549 cells and HeLa cells. In addition, the HBHA protein activated the mitogen-activated protein kinase (MAPK) and nuclear factor kappa B (NF-κB) signaling pathways and promoted the production of tumor necrosis factor-α (TNF-α), interleukin-6 (IL-6), and IL-10 in macrophages. HBHA-mediated TNF-α production was dependent on the activation of the c-Jun N-terminal kinase (JNK) signal pathways, and the IL-6 and IL-10 production was dependent on the activation of extracellular regulated kinase (ERK) 1/2, MAPK p38 (p38), JNK, and nuclear NF-κB signaling pathways. Additionally, the HBHA-mediated activation of innate immunity was dependent on Toll-like receptor 4 (TLR4). Taken together, these results indicate that *N. cyriacigeorgica* HBHA not only adheres to epithelial cells and may be involved in organ colonization, but also plays a critical role in the modulation of innate immunity through the MAPK and NF-κB signaling pathways via TLR4.

## Introduction

*Nocardia* are partially acid-fast, catalase-positive, and Gram-positive bacteria that are widely found in soil and decompose vegetation; they are also found in both fresh- and saltwater (Fatahi-Bafghi, 2018; Churgin et al., 2019). Nocardiosis is usually an opportunistic infection and may cause life-threatening disseminated infections, especially in immunosuppressed hosts (Ambrosioni et al., 2010). Currently, there are more than 90 *Nocardia* species that have been recognized, and ~33 species can cause nocardiosis in humans (Bernardin Souibgui et al., 2017; Churgin et al., 2019). *Nocardia* infection mainly causes brain, lung, and/or skin abscesses, and by dissemination, it can also cause infection in almost all organs; however, the specific mechanism of dissemination remains unclear. The mortality rates of pulmonary nocardiosis are 14–40% (Cooper et al., 2014). The incidence of nocardial infections has increased, accompanying the increase in the number of immunocompromised individuals in the population, and this number has also increased due to improvements in the isolation and molecular identification of *Nocardia* (Gomes et al., 2019).

*Nocardia cyriacigeorgica, Nocardia nova*, and *Nocardia farcinica* are the most likely species to cause disseminated infections, which especially occur in immunosuppressed hosts. *N. cyriacigeorgica* bacteremia is responsible for the most serious cases of infections in humans due to its ability to infect almost all organs, which frequently leads to disease progression despite targeted therapies (Wilson, 2012). However, the cellular and notably the molecular mechanisms by which *N. cyriacigeorgica* causes disseminated infections remain poorly understood. The antigen of heparin-binding hemagglutinin (HBHA), which was initially identified in *Mycobacterium tuberculosis* and *Mycobacterium bovis*, plays a critical role in adhesion to epithelial cells (Menozzi et al., 1998; Lefrancois et al., 2013). Additionally, as a virulence factor in *M. tuberculosis*, HBHA is involved in the dissemination of *Mycobacterium* from the primary infection (Pethe et al., 2001). However, the role of *N. cyriacigeorgica* HBHA in its interaction with host cells remains unknown. By analyzing the *N. cyriacigeorgica* genome sequence, we found a putative HBHA that is similar to *M. tuberculosis* HBHA. We hypothesized that the putative HBHA from *N. cyriacigeorgica* has a similar function to that of the *M. tuberculosis* HBHA.

Additionally, *M. tuberculosis* HBHA has been shown to elicit effective host immune responses against the host (Parra et al., 2004). To further study the function of *N. cyriacigeorgica* HBHA, we investigated the role of this protein in modulating innate immune responses. Macrophages, which are the first line of defense against infection and recognize pathogens, are essential in the regulation of innate immunity, and innate immunity plays a critical role in early defense against *Nocardia* species (Rieg et al., 2010). Additionally, the outcome of nocardiosis is closely related to innate defense mechanisms, especially in the killing and elimination by neutrophils and macrophages (Rieg et al., 2010). The mitogen-activated protein kinase (MAPK) and nuclear factor kappa B (NF-κB) signaling pathways, which are involved in cellular regulation, play essential roles in innate immunity by modulating the production of inflammatory cytokines, such as tumor necrosis factor-α (TNF-α), interleukin-6 (IL-6), IL-10, and IL-1β (Jia et al., 2015). The innate immune system plays a critical role in nocardiosis since the evasion of *Nocardia* spp. was shown to be mediated via uptake and intracellular survival in macrophages and the production of TNF-α after stimulation of blood monocytes is known to be of importance for the innate immune response to *Nocardia* spp. (Kontogiorgi et al., 2013). Toll-like receptor (TLR) proteins activate signal transduction cascades that sequentially engage myeloid differentiation factor 88 (MyD88) and ultimately trigger the MAPK and NF-κB signaling pathways (Jung et al., 2006). Some effectors, such as inflammatory cytokines and chemokines that play critical roles in the innate immune response, are expressed after TLR signal transduction (Jung et al., 2006). *Nocardia erythropolis* cholesterol oxidase is able to activate the MAPK signaling pathway and promote the expression of IL-10 via TLR2 (Bednarska et al., 2014).

In the present study, we investigated the role of *N. cyriacigeorgica* HBHA in its interaction with epithelial cells and macrophages. We analyzed the adhesion of HBHA to A549 cells and HeLa cells. We then assessed the role of *N. cyriacigeorgica* HBHA in organ colonization by constructing an *hbha* deletion mutant (Δ*hbha*) in *N. cyriacigeorgica*. As far as we know, this is the first time that *N. cyriacigeorgica* HBHA was investigated. We used the purified HBHA to study the mechanism by which HBHA regulates the innate immune system. Our results demonstrated that *N. cyriacigeorgica* HBHA could adhere to A549 cells and HeLa cells and that the C-terminal HBHA fragment is responsible for the adhesion function. The deletion of the *hbha* gene in *N. cyriacigeorgica* impaired adhesion to epithelial cells. We also found that HBHA may be involved in *N. cyriacigeorgica*-disseminating infections. The results of the present study on the interaction between *N. cyriacigeorgica* HBHA and macrophages indicate that HBHA may stimulate macrophages to produce cytokines through the activation of NF-κB and MAPK pathways via TLR4. In addition, the production of TNF was dependent on the activation of the c-Jun N-terminal kinase (JNK) signaling pathway, and the extracellular regulated kinase 1/2 (ERK1/2), p38, JNK, and NF-κB signaling pathways were critical to the HBHA-induced production of IL-6 and IL-10.

## Materials and Methods

### Ethics Statement

Laboratory animal care and experimentation were conducted in accordance with animal ethics guidelines. The animal experiments were approved by the Ethics Review Committee of the National Institute for Communicable Disease Control and Prevention at the Chinese Center for Disease Control and Prevention.

### Bacterial Strains, Cells, and Mice

The standard strain of *N. cyriacigeorgica* GUH-2 was purchased from the German Resource Centre for Biological Materials and was grown at 37°C in brain heart infusion (BHI) medium (Oxoid, China). *Escherichia coli* BL21 (DE3) cells (TransGen Biotech, Beijing, China) were grown in LB medium at 37°C. Human monocytic THP-1 cells and murine macrophage RAW 264.7 cells were maintained in our laboratory. THP-1 cells were grown in RPMI 1640 medium (Gibco, Gaithersburg, MD, USA), and RAW 264.7 cells were maintained in DMEM (Gibco) supplemented with 10% fetal bovine serum (FBS; Gibco), 100 IU/ml of penicillin G (Gibco), and 100 μg/ml of streptomycin (Gibco) at 37°C in a humidified incubator with 5% CO_2_. The THP-1 cells were differentiated into macrophages by incubation with 100 ng/ml PMA (Sigma, Germany) for 24 h. Wild-type C57BL/6 mice were purchased from SPF Biotechnology (Beijing, China).

### Plasmids, Reagents, and Antibodies

The pET30a vector (in our laboratory) was used as an expression vector for *N. cyriacigeorgica* GUH-2 HBHA in *E. coli*. The plasmid pK18mobsacB (in our laboratory) was used to construct the *hbha* deletion mutant. Anti-TLR4 (NB100-56727, Novus Biological, USA), anti-TLR2 (NB100-56726, Novus Biological, USA), and IgG_2A_ isotype control (MAB003, R&D, USA) were used in the present study. The following antibodies were purchased from Cell Signaling Technology (Danvers, USA): anti-p-Jnk (4668), anti-p-ERK1/2 (4370), anti-p-p38 (4511), anti-p-p65 (3033), and anti-β-actin (7074). The following pharmacological inhibitors were purchased from Sigma-Aldrich (St. Louis, MO, USA): p38 (SB203580), ERK1/2 (PD98059), JNK (SP600125), and NF-κB (BAY 11-7082). The endotoxin removal kit was purchased from GenScript (Nanjing, China). Human and mouse TNF-α, IL-6, and IL-10 ELISA kits were used in this study (BD, USA). DAPI (4′,6-diamidino-2-phenylindole, dihydrochloride) and Alexa Fluor® 488 donkey anti-mouse IgG were purchased from ThermoFisher Scientific (Carlsbad, CA, USA). The adjuvant was purchased from Biodrago (Suzhou, China).

### Amino Acid Sequence Analysis

The amino acid identities of *N. cyriacigeorgica* GUH-2 HBHA and *M. tuberculosis* HBHA were determined using MEGA (5.05).

### HBHA Protein Preparation

**Figure 1:**
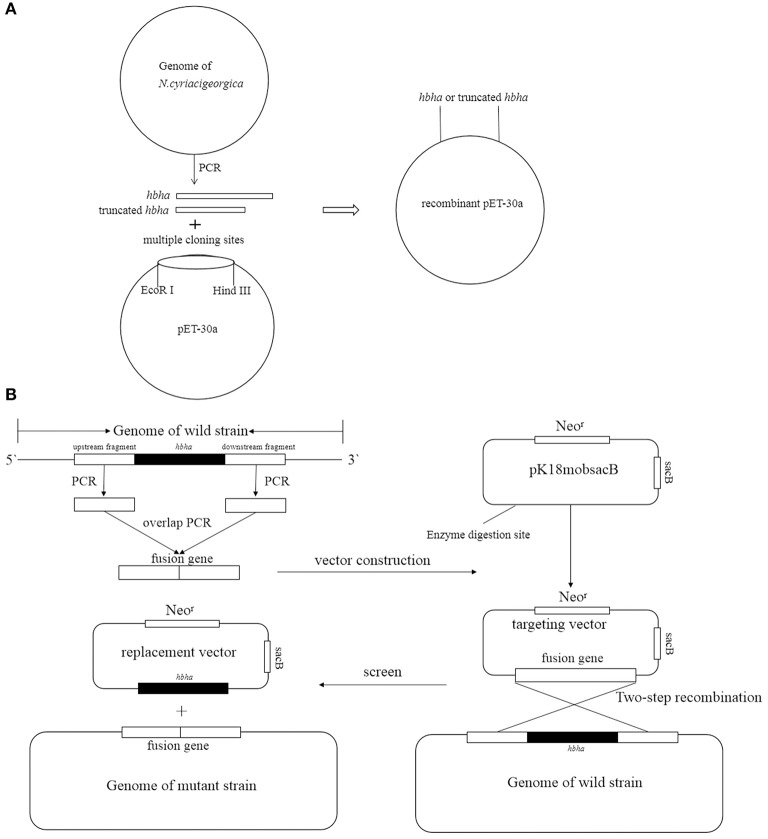
Strategy of construction recombinant pET30a **(A)** and deletion *hbha* gene in *N. cyriacigeorgica* **(B)**.

The *hbha* gene was PCR-amplified using *N. cyriacigeorgica* GUH-2 DNA as a template with the primers shown in Table 1 (hbha-F and hbha-R), and the truncated *hbha* gene lacking the C-terminal domain (38aa) was amplified with the primers Thbha-F and Thbha-R (Table 1). The PCR products (hbha, 678 bp; truncated *hbha*, 561 bp) were digested by *EcoR I* and *Hind III* and then introduced into the pET-30a (+) to generate pET30a-*hbha* or pET30a-truncated hbha, and the recombinant plasmid was sequenced and then transformed into *E. coli* BL21 cells. A flowchart for constructing a recombinant pET30a-HBHA expression plasmid is shown in the Figure 1A. These BL21 cells were grown on LB agar supplemented with 50 μg/ml kanamycin (TransGen Biotech). Recombinant *E. coli* BL21 cells were induced with 0.2 mM of isopropyl β-D-1-thiogalactopyranoside (IPTG, TransGen Biotech) for 6 h at 30°C in LB medium containing 50 μg/ml kanamycin. The bacteria were then lysed by sonication and centrifuged at 12,000 rpm at 4°C for 20 min, and the supernatant was collected. The recombinant proteins were then purified using the His·Bind Purification Kit (Novagen, Germany) in accordance with the manufacturer's instructions. Endotoxin was removed from the purified HBHA proteins using the ToxinEraserTM Endotoxin Removal Kit (GenScript, China). The concentrations of proteins were measured by a bicinchoninic acid (BCA) assay, and the proteins were stored at −80°C until further use. The HBHA protein and its truncated version (about 4 μg) were identified by SDS-PAGE and western blotting with anti-his and HBHA antibodies. The anti-HBHA serum was prepared according to the following steps: (1) The calf muscle of the hind leg of the mouse was injected with 20 μg of HBHA protein (50 μl) with an equal volume of adjuvant. (2) The mice were immunized again after 3 weeks. (3) The anti-serum was collected 2 weeks after the second immunization, and the titer of anti-serum was detected using ELISA.

**Table 1:** Primers used for PCR and RT-PCR.

**Primer**	**Sequence (5^**′**^ to 3^**′**^)**
Dhbha-1F	TCAGGAATTCATCTCCAGCACGACGAGGCG
Dhbha-1R	GGATGCGGCAGGCGAATCATGTTTCGTTCTCCTGGCGT
Dhbha-2F	CAGGAGAACGAAACATGATTCGCCTGCCGCATCCGTTG
Dhbha-2R	GATCAAGCTTGCGTTCTGGTAGGTGCGTGA
Dhbha-F	GCCGAAAGCCAGCAGCCACA
Dhbha-R	CAGAAGGTGAGTGATGGCAGA
hbha-F	TCAGGAATTCACCGACAACGCCACCACCA
hbha-F	GATCAAGCTTTCAAGCCTTCTTCGCGGCGGT
Thbha-F	TCAGGAATTCACCGACAACGCCACCACCA
Thbha-R	GATCAAGCTTCGCGGTCTCGGTGGTGACC
GAPDH-F	AACGACCCCTTCATTGAC
GAPDH-R	TCCACGACATACTCAGCAC
TNF-F	ATGAGCACAGAAAGCATGATC
TNF-R	TACAGGCTTGTCACTCGAATT
IL-6-F	GTTCTCTGGGAAATCGTGGA
IL-6-R	TGTACTCCAGGTAGCTA
IL-10-F	TGCTAACCGACTCCTTAATGCAGGAC
IL-10-R	CCT TGATTTCTGGGCCATGCTTCTC

### Construction of Deletion Mutants

The *hbha* in-frame deletion mutant (Δ*hbha*) was constructed by homologous recombination. A diagram describing the generation of a deletion hbha in *N. cyriacigeorgica* is shown in Figure 1B. An upstream DNA fragment (1,060 bp with an *EcoRI* site) and downstream DNA fragment (709 bp with a *HindIII* site) of *hbha* were amplified by PCR using specific primers (Dhbha-1F/Dhbha-1R and Dhbha-2F/Dhbha-2R; Table 1). These two fragments were ligated by bypass PCR amplification using specific primers (Dhbha-1F/Dhbha-2R; Table 1) to generate the *hbha* deletion fragment, which was subsequently inserted into the suicide plasmid pk18mobsacB. Logarithmic-phase *N. cyriacigeorgica* were washed three times with ice-cold water and were then resuspended in ice-cold 10% glycerol to prepare competent cells (Ishikawa et al., 2006). The recombinant pK18mobsacB plasmid was transformed into competent *N. cyriacigeorgica* by electroporation using standard settings (i.e., 2.5 kV, 1,000 Ω, and 25 μF). After pulsing with a Gene Pulser Xcell apparatus (Bio Rad, USA), the *N. cyriacigeorgica* were added to 900 μl of BHI broth and incubated for 2 h while shaking at 37°C. The transformed cells were then plated onto BHI agar plates containing 100 μg/ml of neomycin and incubated at 37°C for 2 days. The positive colonies were selected and plated onto BHI agar plates containing 20% sucrose to select for the cells that did not contain the genome-integrated *sacB*-containing plasmid. The deletion of *hbha* was confirmed by PCR and DNA sequencing using the Dhbha-F and Dhbha-R primer pair (Table 1).

### Infection of Mice With *N. cyriacigeorgica* and the Δ*hbha* Mutant Strain

The clones of wild-type and mutant strains on BHI agar were scraped into BHI media and cultured at 37°C with shaking overnight, and the bacteria were then cultured in fresh BHI media with a dilution of 1:100 for 18 h. The bacteria were washed three times with PBS and were then adjusted to 3 × 10^8^ CFU/ml. Intraperitoneal injection was performed with 3 × 10^7^ viable units of parental or mutant strains. At 1 and 4 days after infection, nine mice per group were sacrificed, and individual lungs, brains, and spleens were removed and homogenized. Serial dilutions were plated onto BHI agar plates for colony counting, and the numbers of colonies in different organs were determined.

### Interactions of HBHA With A549 Cells and HeLa Cells

Briefly, approximately 2 × 10^5^ A549 cells or HeLa cells were seeded into a 24-well plate with glass coverslips and were grown until they were confluent. For immunofluorescent assays, 10 μg of the HBHA protein or truncated HBHA protein was added to cell monolayers. After 1 h of incubation, cells were thoroughly washed three times with PBS to remove non-adhesive protein. Then, cell monolayers were fixed with 4% paraformaldehyde for 15 min and were treated with 0.1% triton for 10 min. Subsequently, anti-HBHA serum or control serum was added to the cells at a dilution of 1:500, and the cells were incubated for 30 min at 37°C, after which the cells were washed three times with PBS. Alexa Fluor® 488 donkey anti-mouse IgG was then added to the cell monolayers at a dilution of 1:2,000, and the cells were incubated for 30 min at 37°C. DAPI was added to the cells at a dilution of 1:1,000 for 10 min. Finally, the cells were imaged with a fluorescent microscope (Echo Revolve, USA).

### Adhesion of A549 Cells and HeLa Cells by *N. cyriacigeorgica* GUH-2

*Nocardia cyriacigeorgica* GUH-2 wild-type and Δhbha strains were used to further confirm the adhesion function of HBHA. A549 or HeLa cells were grown in complete DMEM medium at an initial density of 2 × 10^5^ cells/well in 24-well plates. The cells were infected with the wild-type or the Δhbha strain of *N. cyriacigeorgica* for 1 h at a multiplicity of infection (MOI) of 10. After incubation, the cells were washed three times with sterile PBS and then solubilized with 1 ml of sterile water supplemented with 0.1% triton x-100 for 20 min. The numbers of bacteria were determined by plating serially diluted cultures on BHI plates.

### Cytokine ELISAs

RAW264.7 cells or THP-1 cells were seeded onto 24-well plates at a density of 2 × 10^5^ cells/well and were stimulated with HBHA for 18 h. Before stimulation with HBHA, THP-1 cells were differentiated into macrophages by incubation with 100 ng/ml PMA for 24 h. To exclude the contamination of lipopolysaccharide (LPS) that may cause cell stimulation in the purified HBHA, HBHA was preincubated with polymyxin B (a specific inhibitor for LPS, INALCO, USA) for 1 h at 37°C. To determine the specificity of activation and the chemical nature of HBHA, HBHA was pretreated with proteinase K (50 μg/ml; Sigma) for 24 h at 37°C, followed by heating for 10 min at 98°C to inactivate the enzyme. To block MAPK and NF-κB signaling, RAW264.7 cells were pretreated with inhibitors of p38 (10 μM), ERK (20 μM), JNK (20 μM), or NF-κB (0.5, 1, or 2 μM) for 1 h at 37°C prior to the HBHA stimulation described above. In experiments designed to block TLR signaling, THP-1 cells were pretreated with antibodies against human TLR2 (10 μg/ml), TLR4 (10 μg/ml), or an IgG isotype-matched control antibody (10 μg/ml) for 1 h at 37°C. The cell culture supernatants were then collected at 18 h post-stimulation, and the levels of cytokines were measured using ELISA.

### Western Blotting

RAW264.7 cells or THP-1 cells were seeded onto six-well plates at a density of 1 × 10^6^ cells/well. After stimulating with HBHA for the indicated time, the cells were lysed with lysis buffer supplemented with phosphatase and protease inhibitors (CWBIO, Beijing, China) on ice. The total protein in the supernatant was collected after centrifugation at 12,000 × g at 4°C for 25 min. Equal protein concentrations were separated by SDS-PAGE and were then transferred to polyvinylidene fluoride membranes (Millipore). After blocking with 5% nonfat dry milk in TBST for 2 h at room temperature, the membranes were washed three times with TBST and were then incubated overnight with primary antibodies at 4°C, including p-ERK1/2, p-p38, p-JNK, NF-κB p-p65, and β-actin, according to the manufacturer's instructions. After washing with TBST, membranes were incubated for 1 h with HRP-conjugated anti-rabbit IgG (Beyotime Biotechnology), and the bands were measured by the Western Lightning Plus ECL kit (PerkinElmer, USA).

### RT-PCR Analysis

Total RNA was extracted from RAW264.7 cells that were stimulated with HBHA using the RNeasy Plus Mini Kit (Qiagen, Hilden, Germany) according to the manufacturer's recommended instructions. Potential gDNA contamination was removed during the process of extraction. Then, cDNA was synthesized at 37°C for 15 min, followed by 85°C for 5 min, using the PrimeScript RT reagent kit (TaKaRa, Kusatsu, Japan). Real-time PCR was carried out using the SYBR Premix Ex Taq II reagents (TaKaRa) according to the manufacturer's protocol. RNA levels of the analyzed genes were normalized to the amount of GAPDH in each sample. The primers (Table 1) of TNF-α, IL-6, IL-10, and GAPDH were synthesized by Sangon (Shanghai, China). The RT-PCR experiments were conducted across three independent experiments.

### Statistical Analysis

Results are shown as the mean ± standard deviation (SD) of triplicate experiments. Analyses were performed using SPSS 22.0. Group means and SDs were compared with Student's *t*-tests. A *P* < 0.05 was considered to be statistically significant.

## Results

### Expression and Purification of *N. cyriacigeorgica* HBHA Protein

**Figure 2:**
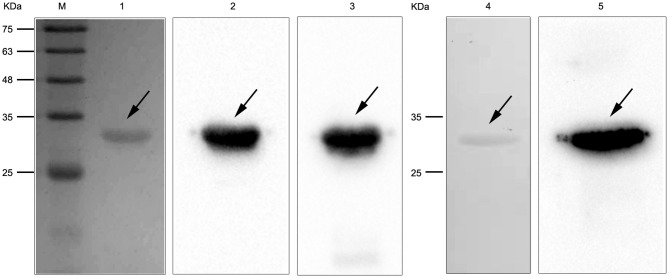
SDS-PAGE and western blotting of *Nocardia cyriacigeorgica* heparin-binding hemagglutinin HBHA protein. Lane M: protein marker. Lane 1: Purified HBHA protein. Lane 2: HBHA protein was detected with anti-HBHA sera. Lane 3: HBHA protein was detected with anti-His sera. Lane 4: Purified truncated HBHA protein. Lane 5: Truncated HBHA protein was detected with anti-HBHA sera.

To study the interaction of *N. cyriacigeorgica* HBHA with host cells, the recombinant HBHA protein and the truncated HBHA protein were expressed in *E. coli*. DNA sequencing confirmed that the recombinant vector was constructed successfully. The recombinant HBHA protein and truncated protein were mainly expressed in the supernatant, which indicated that the recombinant protein was soluble in BL21. SDS-PAGE showed that the purified protein presented as a single band, and western blotting revealed that the recombinant HBHA protein and truncated HBHA protein were each specifically recognized by antibodies of anti-HBHA and anti-His, respectively (Figure 2).

### HBHA Is Involved in Bacterial Adhesion to Host Cells

**Figure 3:**
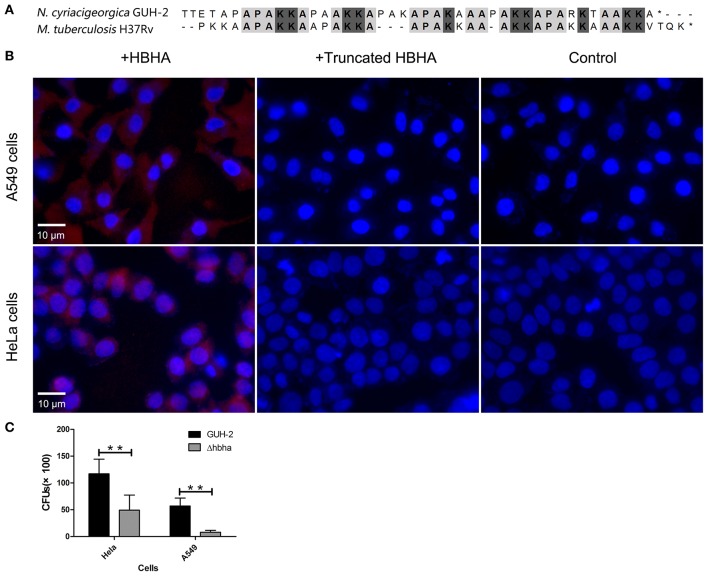
*N. cyriacigeorgica* HBHA adheres to A549 cells and HeLa cells. **(A)** Amino acid sequence alignment between *N. cyriacigeorgica* HBHA and *Mycobacterium tuberculosis* HBHA. **(B)** Immunofluorescent assays demonstrate the adhesion of *N. cyriacigeorgica* HBHA to A549 cells and HeLa cells. HBHA protein or the HBHA protein lacking C-terminal domain (10 μg) was added to A549 cells or HeLa cells separately for 1 h, and the protein adhesion onto the cells was detected with fluorescent microscopy. Data are representative of at least three independent experiments. **(C)** HeLa or A549 cells were infected with wild-type or Δ*hbha* strains for 1 h, and the bacteria adherence to cells was then determined. The assays were performed in triplicate, and the results are expressed as the means ± SD. **P < 0.01.

It has been reported that *M. tuberculosis* HBHA is involved in adhesion to epithelial cells (Lefrancois et al., 2013). Besides, the C-terminal Lys-rich region is responsible for the binding of *M. tuberculosis* HBHA to epithelial cells (Menozzi et al., 1998). The results of amino acid sequence alignment showed that *N. cyriacigeorgica* HBHA has a lysine-rich region similar to *M. tuberculosis* HBHA at the C-terminus (Figure 3A). Hence, we hypothesized that *N. cyriacigeorgica* HBHA may also be involved in adhesion to host cells. Immunofluorescent assays showed that HBHA adhered to both HeLa cells and A549 cells, but that the truncated HBHA lacking the C-terminal fragment was barely visible in apposition to HeLa cells or A549 cells, which was a similar phenotype to that of the control group (Figure 3B). These results indicate that *N. cyriacigeorgica* HBHA protein adheres to host cells and that the C-terminal domain of HBHA is critical to adhesion. A Δ*hbha* with an in-frame deletion was constructed to further investigate the role of HBHA during host cell adhesion. Wild-type and Δ*hbha* were used in adhesion assays with HeLa and A549 cells. The results showed that adhesion of the *hbha* mutant to HeLa cells or A549 cells was significantly reduced compared to *N. cyriacigeorgica* wild-type bacteria (*P* < 0.01; Figure 3C). These above results demonstrated that *hbha* is an important component for the adhesion of *N. cyriacigeorgica* to host cells.

### *hbha* May Be Involved in Colonization of *N. cyriacigeorgica* in Organs

**Figure 4:**
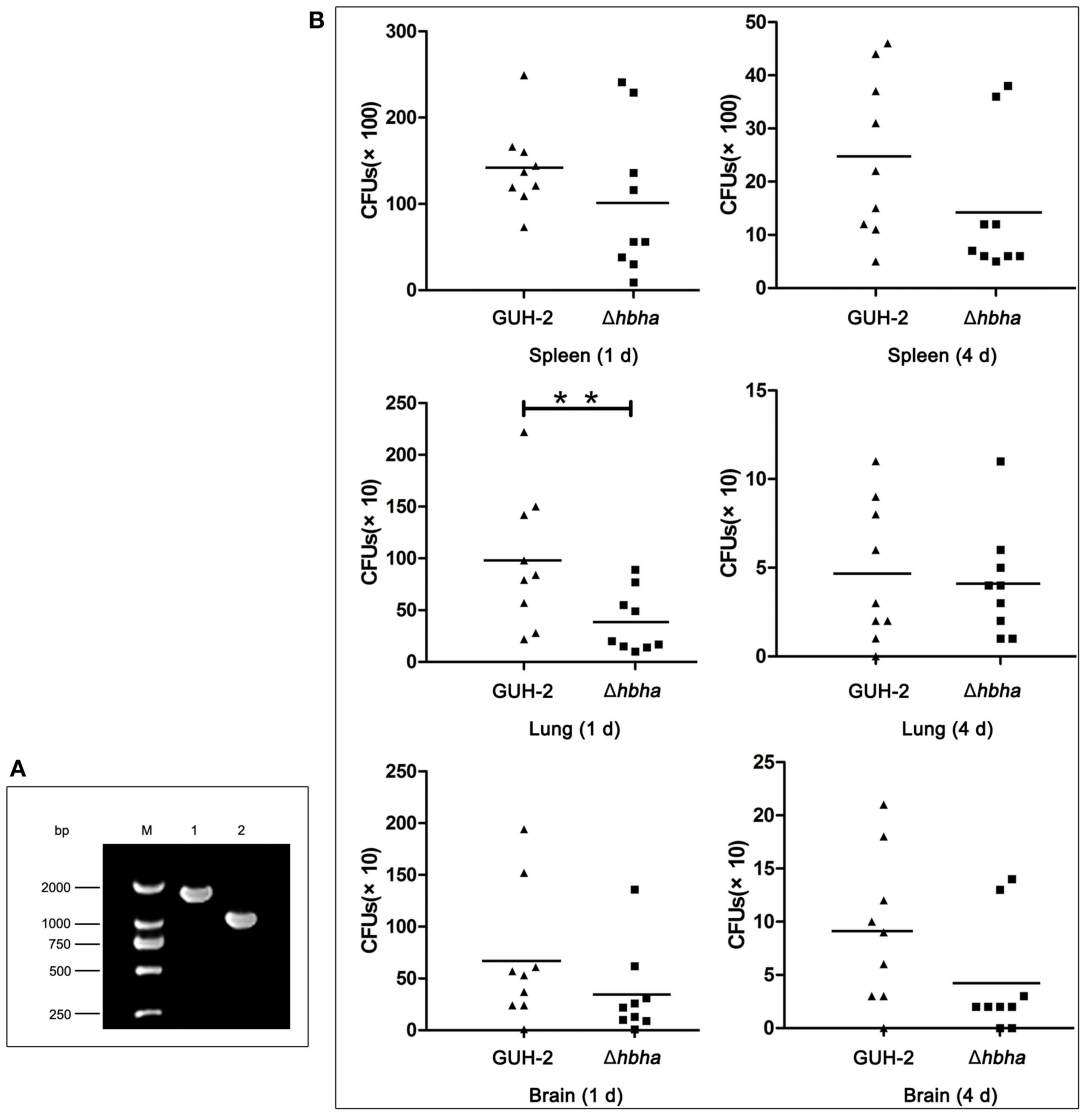
Colonization of *N. cyriacigeorgica* Δ*hbha* mutant and wild-type strains in organs of mice. **(A)** Confirmation of the deletion of the *hbha* gene by PCR. Lane M: DNA marker. Lane 1: Gene amplification fragments from the genome of wild-type *N. cyriacigeorgica*. Lane 2: Gene amplification fragments from the genome of the Δ*hbha* deletion strain. **(B)** Colonization of wild-type strain and Δ*hbha* deletion strain in spleens, lungs, and brains of mice at 1 and 4 days after intraperitoneal infection with the *N. cyriacigeorgica* wild-type or Δ*hbha* mutant strain. The viable units were counted by plating on brain heart infusion (BHI) plates, and the results of each time point are the means and standard deviations of CFUs from nine mice. **P < 0.05.

The above assays showed that *N. cyriacigeorgica* HBHA exhibited an adhesive function. As such, we next hypothesized that HBHA may be involved in colonization in different organs. It has been reported that *M. tuberculosis* HBHA is involved in mycobacterial extrapulmonary dissemination (Pethe et al., 2001), suggesting that HBHA may play a role in colonization in different organs. To verify the function of *N. cyriacigeorgica* HBHA during infection, wild-type and *hbha* mutant strains were used in this assay. First, the *hbha* of *N. cyriacigeorgica* was deleted using markerless in-frame deletion systems, which do not create undesirable polar effects on downstream genes (Merritt et al., 2007). The deletion of *hbha* was verified using PCR. As shown in Figure 4A, and as expected, the size of the *hbha* amplicon was reduced in the mutants compared with the wild-type strain. Additionally, there was no significant difference in the growth curve between the mutant and wild-type strains (data not shown), indicating that the deletion of the gene did not affect the growth ability of the strain. To study the role of this gene in the colonization of different organs, nine mice each group were infected intraperitoneally with the wild-type and Δ*hbha* strains; this route of infection allows bacteria to enter the bloodstream quickly. On the first and fourth days after infection, the lungs, spleens, and brains of the 36 mice were harvested and ground, and the numbers of colonies in the tissues were counted by plating serially diluted tissue samples on BHI plates. As shown in Figure 4B, the lung, spleen, and brain colonization profiles of *N. cyriacigeorgica* Δ*hbha* show fewer colonies than those of the wild-type strains. These results indicate that HBHA may play a role in colonization in different organs for *N. cyriacigeorgica* GUH-2 during the infection process.

### *N. cyriacigeorgica* HBHA Activates the MAPK and NF-κB Signaling Pathways

**Figure 5:**
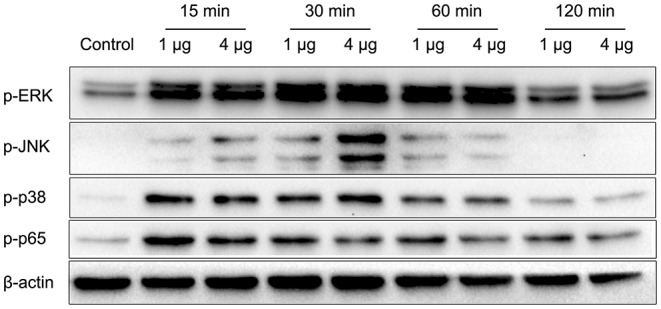
HBHA promotes the phosphorylation of extracellular regulated kinase (ERK), c-Jun N-terminal kinase (JNK), p38, and P65. RAW264.7 cells were stimulated with HBHA (1 and 4 μg/ml) for 15–120 min. Protein extracts were prepared, and the phosphorylation status of ERK1/2, p38, JNK, and p65 was detected via western blotting. Data are representative of at least three independent experiments.

*Nocardia cyriacigeorgica* not only invades and replicates within epithelial cells but also interacts with macrophages. In the above experiments, we demonstrated an adhesive function of HBHA. To understand the other functions of HBHA more comprehensively, we further studied the role of HBHA in innate immune regulation. Many innate immune responses involve the MAPK and NF-κB signaling pathways. To investigate whether HBHA regulates these two signaling pathways, we detected the effect of HBHA on macrophages using western blotting. RAW264.7 cells were stimulated with HBHA (1 and 4 μg/ml), and the phosphorylation statuses of p38, JNK, ERK1/2, and p65 were detected using western blotting at different time points. The results showed that stimulation with HBHA resulted in rapid phosphorylation of p38, ERK1/2, and p65 at 15 min after stimulation, as well as phosphorylation of JNK at 30 min after stimulation (Figure 5). The phosphorylations of p38, JNK, ERK1/2, and p65 returned to baseline after ~2 h of stimulation with HBHA. These results indicate that HBHA plays a role in activating innate immune signaling pathways.

### HBHA Stimulates the Production of TNF-α, IL-6, and IL-10 in RAW264.7 Macrophages

**Figure 6:**
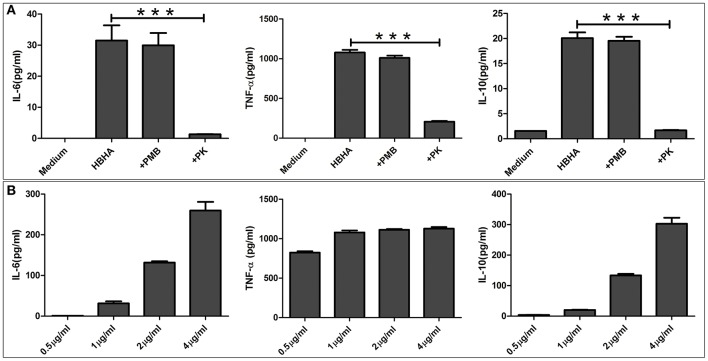
HBHA induces interleukin-6 (IL-6), tumor necrosis factor-α (TNF-α), and IL-10 production in RAW264.7 cells. **(A)** RAW264.7 cells were stimulated with HBHA (1 μg/ml) for 18 h at 37°C, and the cytokines from culture supernatants were measured by ELISA. To exclude the possibility that lipopolysaccharide (LPS) contaminated the recombinant protein, HBHA was pretreated with an inhibitor of LPS, polymyxin B (+PMB). To confirm that the activation of RAW264.7 cells was attributed to HBHA, the protein (1 μg/ml) was pretreated with proteinase K. IL-6, TNF-α, and IL-10 in the culture supernatants was measured at 18 h. Data are expressed as the mean ± SD from three separate experiments. ***P < 0.001. **(B)** RAW264.7 cells were cultured with various concentrations of HBHA (0.5, 1, 2, or 4 μg/ml). Cell culture supernatants were collected at 18 h, and the levels of IL-6, TNF-α, and IL-10 were detected with ELISA.

Since the results of the above assays showed that HBHA activated the innate immune signaling pathway, we next tested downstream immunologically relevant effector factors, such as TNF-α, IL-6, and IL-10. RAW264.7 macrophages were stimulated with various concentrations of HBHA for 18 h, and the levels of TNF-α, IL-6, and IL-10 in the culture supernatants were measured by ELISA. HBHA stimulated RAW264.7 cells to upregulate the expression of TNF-α, IL-6, and IL-10, and the expression of IL-6 and IL-10 were upregulated in a dose-dependent manner (Figure 6B). To exclude the possibility of contamination of LPSs that may stimulate cytokine production in these cells, HBHA was pretreated with polymyxin B. Our results showed that polymyxin B did not alter the HBHA-induced production of TNF-α, IL-6, and IL-10 (Figure 6A). Furthermore, cytokine production was abrogated when macrophages were stimulated with HBHA pretreated with proteinase K, which indicated that the effect was specifically attributed to HBHA.

### HBHA-Induced Increases in TNF-α, IL-6, and IL-10 Expression Depend on the Activation of MAPK and NF-κB Signaling Pathways

**Figure 7:**
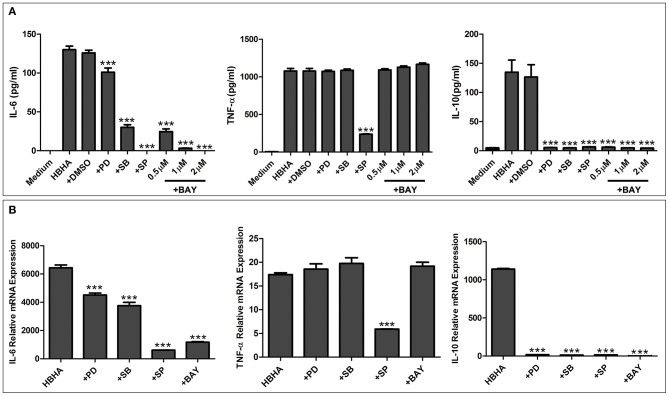
HBHA-mediated cytokine secretion is dependent on mitogen-activated protein kinase (MAPK) and nuclear factor kappa B (NF-κB) signaling pathways at both the protein and mRNA levels. **(A)** RAW264.7 cells were pretreated with PD98059 (+PD; 20 μM), SB203580 (+SB; 10 μM), SP600125 (+SP; 20 μM), BAY 11-7082 (+BAY; 0.5, 1, and 2 μM), or DMSO (0.01%) and then induced with HBHA (1 μg/ml). IL-6, TNF-a, and IL-10 in supernatants were measured by ELISA after incubation for 18 h. **(B)** RAW264.7 cells were pretreated with PD98059 (+PD; 20 μM), SB203580 (+SB; 10 μM), SP600125 (+SP; 20 μM), or BAY 11-7082 (+BAY; 2 μM) and were then induced with HBHA (1 μg/ml) for 6 h. The relative mRNA levels of IL-6, TNF-α, and IL-10 were calculated and expressed as the fold changes relative to that of the control. Data are expressed as the mean ± SD from three separate experiments. ***P < 0.001.

To further elucidate the functional roles of JNK, p38, ERK1/2, and NF-κB in HBHA-induced cytokine production, RAW264.7 cells were pretreated with the following specific inhibitors: 20 μM PD98059 (MEK inhibitor), 20 μM SP600125 (JNK inhibitor), 10 μM SB203580 (p38 MAPK inhibitor), or 0.5, 1, or 2 μM BAY 11-7082 (NF-κB inhibitor) for 1 h at 37°C. Then the cells were treated with HBHA (1 μg/ml), and the TNF-α, IL-6, and IL-10 production was measured by ELISA after 18 h. As shown in Figure 7A, IL-6 production was significantly inhibited by the p38, JNK, and NF-κB inhibitors and was slightly inhibited by ERK1/2 inhibitors, which indicated that HBHA-induced expression of IL-6 was dependent on the activation of the ERK1/2, p38, JNK, and NF-κB signaling pathways. TNF-α expression was significantly inhibited by the JNK inhibitor, indicating that activation of the JNK signaling pathway was critical to the HBHA-induced TNF-α production. IL-10 production was significantly inhibited by the ERK1/2, p38, JNK, and NF-κB inhibitors, which indicated that HBHA-induced expression of IL-10 was dependent on the activation of ERK1/2, p38, JNK, and NF-κB signaling pathways. Similar results were obtained when the relative mRNA expressions of TNF-α, IL-6, and IL-10 were assessed (Figure 7B). These results indicate that the MAPK and NF-κB signaling pathways play a critical role in HBHA-induced inflammatory cytokine production.

### HBHA Stimulates the Production of Cytokines and the Activation of MAPK and NF-κB Signaling Pathways via a TLR4-Dependent Mechanism

**Figure 8:**
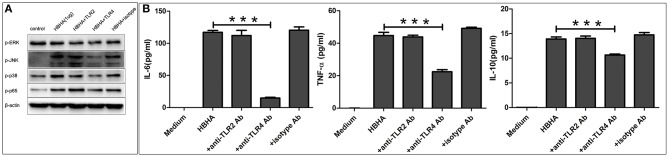
Toll-like receptor 4 (TLR4) is required for the HBHA-stimulated activation of MAPK and NF-κB signaling pathways, and the secretion of cytokines in macrophages. **(A)** THP-1 cells were pretreated with anti-TLR2 (10 μg/ml), anti-TLR4 (10 μg/ml), or isotype control antibodies (10 μg/ml) for 1 h and were then induced with HBHA for 30 min, and the phosphorylation levels of p38, JNK, ERK1/2, and p65 were detected by western blotting. **(B)** THP-1 cells were pretreated with blocking antibodies for 1 h and were then stimulated with HBHA. The levels of IL-6, TNF-α, and IL-10 in the supernatants were measured by ELISA after 18 h. Data are expressed as the mean ± SD from three independent experiments. ***P < 0.001.

To determine the role of TLR in HBHA-induced immune activation, THP-1 cells were pretreated with or without the blocking antibody of human TLR2, TLR4, or an isotype control antibody for 1 h prior to treatment with HBHA. The phosphorylation levels of p38, JNK, ERK1/2, and p65 induced by HBHA were detected using western blotting at 30 min after stimulation. The production of TNF-α, IL-6, and IL-10 were measured using ELISA at 18 h after HBHA induction. LPS and Pam3CSK4 were used as positive controls to verify the effects of the blocking antibody (data not shown). As shown in Figure 8A, the HBHA-induced phosphorylation of p38, JNK, ERK1/2, and p65 were attenuated when the THP-1 cells were preincubated with anti-TLR4 antibodies, whereas the phosphorylation statuses of p38, JNK, ERK1/2, and p65 were not affected by preincubation with anti-TLR2 or the isotype control antibody. As shown in Figure 8B, The HBHA-induced increases in the production of TNF-α, IL-6, and IL-10 were attenuated by anti-TLR4 antibodies, whereas neither anti-TLR2 nor isotype control antibodies affected the HBHA-induced increase in cytokine production. Therefore, the activation of MAPK and NF-κB signaling pathways and the production of TNF-α, IL-6, and IL-10 in response to HBHA are mediated by TLR4.

## Discussion

The intracellular pathogenic bacteria *N. cyriacigeorgica* has been recognized as an emerging pathogenic entity around the world (Schlaberg et al., 2008). However, the pathogenic mechanism and virulence factors of this bacterium are still unclear. The virulence factors of HBHA from *M. tuberculosis* play a critical role in the infection process (Zheng et al., 2017). Through genomic analysis, *hbha* genes are known to be widely distributed among Gram-positive Actinobacteria as a single copy gene, such as in *Nocardia, Rhodococcus*, and *Mycobacterium* genera. HBHA proteins have been thoroughly studied for *Rhodococcus opacus* and *M. tuberculosis*, and their functions are different in different types of cells (Lanfranconi and Alvarez, 2016). It has been found that a putative *hbha* gene also exists in the genome of *N. cyriacigeorgica*. However, there is currently no relevant published study on the function of the *hbha* gene in *N. cyriacigeorgica*. Therefore, we investigated the possible role of *N. cyriacigeorgica hbha* in the infection process, especially in terms of the interaction between HBHA and epithelial cells or macrophages.

To cause infection, pathogenic microorganisms need to adhere to target tissues or host cells by interacting with a variety of cell surface receptors (Biet et al., 2007). As far as we know, there are few previous studies on adhesion-related virulence factors of *Nocardia*. The HBHA protein from *M. tuberculosis* plays an important role in the interaction with host cells during the infection process (Delogu and Brennan, 1999). Additionally, the C-terminal domain of HBHA from *M. tuberculosis* is required to adhere to the surface of host cells (Pethe et al., 2000). In the present study, the complete and truncated genes of *N. cyriacigeorgica hbha* were expressed, and the two respective recombinant proteins could be identified specifically by anti-His or anti-HBHA antibodies (Figure 2). To determine whether HBHA exhibited adhesion properties and to isolate the adhesion domain of this protein, immunofluorescent assays were performed. As shown in Figure 1, the HBHA protein adhered to A549 cells and HeLa cells, but the truncated HBHA, which did not contain the C-terminal domain, was not detected on these two epithelial cells, which indicated that the *N. cyriacigeorgica* HBHA has an adhesive function and that the functional region is located at the C-terminus domain that is composed of lysine-rich motifs. When we used Δ*hbha* strains and wild-type strains to further study the difference in the number of *N. cyriacigeorgica* cells that adhered to A549 cells and HeLa cells, we found a significant difference (Figure 3C). The HBHA protein (called TadA in rhodococci) in *R. opacus* PD630 is involved in lipid body formation and maturation (MacEachran et al., 2010). A recent study showed that the *M. tuberculosis* HBHA also participates in the generation of intracytosolic lipid (ILI) inclusions and that the truncated HBHA lacking the C-terminal heparin-binding domain is no longer able to induce ILI accumulation (Raze et al., 2018). From current research on this protein, HBHA is believed to have mainly two functions. The present study focused on the *N. cyriacigeorgica* HBHA adhesive function; thus, whether HBHA is also related to liposome formation requires further research. In *in vivo* assays, we found that the numbers of the wild-type strain in different organs of mice and at different times post-infection were higher than those of the Δ*hbha* strain, which indicated that *N. cyriacigeorgica hbha* may play an important role during infection and may be involved in *N. cyriacigeorgica* colonization of different organs. It has been reported that the heparin-binding domain structure of mycobacterial HBHA can adapt upon interaction with glycosaminoglycans, which may be involved in the binding specificities to hosts or organs (Neyrolles et al., 2012).

We also found that the *N. cyriacigeorgica* HBHA adhered to non-phagocytic cells and may have been involved in the colonization of different organs, which suggests that it may play an important role during infection as a virulence factor. To fully characterize the role of *N. cyriacigeorgica* HBHA during infection, especially in the regulation of innate immunity, we studied the interaction of HBHA with macrophages. Both innate leukocytes and non-conventional T-lymphocytes are of importance in the defense against *Nocardia asteroides*, and the host will have increased susceptibility to infection with *Nocardia* in the absence of neutrophils (Moore et al., 2000; Tam et al., 2012). Both the MAPK and NF-κB signaling pathways play critical roles in innate immunity and are central to the production of inflammatory cytokines, such as TNF-α, IL-6, and IL-1β (Jia et al., 2015). ERK, JNK, and p38, which belong to the MAPK signaling pathway, have been shown to regulate the production of inflammatory cytokines independently or simultaneously (Lee et al., 1994; Luo et al., 2010). The phosphorylation of p65, which is associated to the NF-κB signaling cascade, is of importance for innate immunity since it also stimulates the production of cytokines (Valentinis et al., 2005). In the present study, we found that *N. cyriacigeorgica* HBHA was capable of activating the MAPK and NF-κB signaling pathways by promoting the phosphorylation of ERK, JNK, p38, and p65. Our results showed that *N. cyriacigeorgica* HBHA stimulated RAW264.7 cells to significantly increase the production of both pro-inflammatory cytokines (TNF-α and IL-6) and anti-inflammatory cytokines (IL-10). The complicated underlying mechanism of regulation between pro-inflammatory cytokines and anti-inflammatory cytokines remains unknown, and the biochemical cross talk between the NF-κB and MAPK signaling pathways in HBHA-stimulated expression of TNF-α, IL-6, and IL-10 in macrophages needs to be further clarified in future studies. Solis-Soto et al. reported that a mixture of pro-inflammatory and anti-inflammatory cytokines is secreted at the same time by host cells from mice infected with *Nocardia brasiliensis* (Solis-Soto et al., 2008). Wendling et al. have shown that the Rv1808 protein from *M. tuberculosis* stimulates macrophages to upregulate both pro-inflammatory and anti-inflammatory cytokines (Deng et al., 2014). Reiling et al. reported that *Mycobacterium avium* simultaneously regulates the secretion of anti-inflammatory and pro-inflammatory cytokines by macrophages (Reiling et al., 2001). It has been reported that *N. brasiliensis* regulates macrophage cytokine production, which may contribute to pathogenesis, and the production of IL-10 has also been shown to be increased after infection of mice with *N. brasiliensis* (Salinas-Carmona et al., 2009). In our present study, we analyzed the role of phosphorylated MAPK and NF-κB in triggering the production of TNF-α, IL-6, and IL-10. Highly specific inhibitors were used to suppress the activation of p38, ERK1/2, JNK, and NF-κB kinase cascades. Our results showed that the production of TNF-α was significantly reduced via JNK inhibitors (SP600125) at both the protein and mRNA levels. However, the *M. tuberculosis* HBHA-induced production of TNF-α is regulated by p38, ERK1/2, JNK, and NF-κB signaling pathways (Kim et al., 2011). Additionally, our results showed that p38, ERK1/2, JNK, and NF-κB inhibitors reduced the production of IL-6 and IL-10 at both protein and mRNA levels. These results indicate that the JNK signaling pathway is critical to the *N. cyriacigeorgica* HBHA-induced TNF-α production. Additionally, our results suggest that the production of IL-6 and IL-10 by macrophages stimulated with *N. cyriacigeorgica* HBHA is regulated by the p38, ERK1/2, JNK, and NF-κB signaling pathways.

TLRs, especially TLR2 and TLR4, play an important role in activating MAPK and NF-κB signaling in macrophages. Bacterial components, such as proteins or lipoproteins, stimulate the secretion of inflammatory cytokines by modulating TLR2 or TLR4 signaling (Gilleron et al., 2003; Gehring et al., 2004; Sanchez et al., 2009). Millan-Chiu et al. reported that TLR2 and TLR4 were expressed in tissues of mice infected with *N. brasiliensis* during the early infection stages (Millan-Chiu et al., 2011). In the present study, we found that the HBHA-induced secretion of inflammatory cytokines and activation of MAPK and NF-κB signaling pathways was attenuated by the blocking effect of the anti-TLR4 antibody, which suggested that TLR4 was essential for the activation of MAPK and NF-κB signaling pathways and the production of cytokines by macrophages after stimulation with *N. cyriacigeorgica* HBHA. When the peripheral blood mononuclear cells from patients infected with *Nocardia* were stimulated with TLR4 agonists (LPS) or *Nocardia* antigens, the production of inflammatory cytokines (TNF-α and IL-6) was lower but not after stimulation with TLR2 agonists (Pam3Cys), which may indicate that the main recognition receptor for the *Nocardia* antigen is TLR4 (Kontogiorgi et al., 2013). However, in an earlier study, it has been suggested that TLR2 may play a major role in identifying Gram-positive bacteria (Takeuchi et al., 1999).

Our results demonstrated that *N. cyriacigeorgica* HBHA adhered to A549 cells and HeLa cells and that the C-terminal Lys-rich fragment is essential for the adhesive function. Additionally, *N. cyriacigeorgica* HBHA may play a role in colonizing in different organs during infection. Furthermore, HBHA may regulate the innate immune response by activating the MAPK and NF-κB signaling pathways and stimulating the production of inflammatory cytokines (IL-6, TNF-α, and IL-10) via TLR4. Thus, the present study provides a better understanding of the adhesive and immunoregulatory functions of *N. cyriacigeorgica* HBHA, which may lead to insights in the prevention and treatment of *Nocardia* infection.

## Data Availability Statement

All datasets generated for this study are included in the article/supplementary material.

## Ethics Statement

The animal experiments were approved by the Ethics Review Committee of the National Institute for Communicable Disease Control and Prevention at the Chinese Center for Disease Control and Prevention.

## Author Contributions

XJ and ZL conceived and designed the experiments. XJ and XZ wrote the manuscript and performed the experiments. XJ and LS analyzed the data. LS, XH, HS, LH, SX, HL, XQ, ML, XW, and NZ contributed reagents, materials, and analysis tools. ZL provided financial and administrative support and approved the final version of the manuscript.

### Conflict of Interest

The authors declare that the research was conducted in the absence of any commercial or financial relationships that could be construed as a potential conflict of interest.
